# The “Vertigo” of the Food Sector within the Triangle of Climate Change, the Post-Pandemic World, and the Russian-Ukrainian War

**DOI:** 10.3390/foods12040721

**Published:** 2023-02-07

**Authors:** Charis M. Galanakis

**Affiliations:** 1 Galanakis Laboratories, Research & Innovation Department, 73131 Chania, Greece; charismgalanakis@gmail.com; 2 Food Waste Recovery Group, ISEKI Food Association, 1190 Vienna, Austria

**Keywords:** food security, food resilience, food safety, food waste, bioeconomy, sustainability

## Abstract

Over the last few years, the world has been facing dramatic changes due to a condensed period of multiple crises, including climate change, the COVID-19 pandemic, and the Russian–Ukrainian war. Although different, these consecutive crises share common characteristics (e.g., systemic shocks and non-stationary nature) and impacts (e.g., disruption of markets and supply chains), questioning food safety, security, and sustainability. The current article analyses the effects of the noted crises in the food sector before proposing target mitigation measures to address the different challenges. The goal is to transform the food systems to increase their resilience and sustainability. This goal can only be achieved if all relevant actors within the supply chain (e.g., governments, companies, distributors, farmers, etc.) play their role by designing and implementing target interventions and policies. In addition, the transformation of the food sector should be proactive concerning food safety, circular (valorizing several bioresources under the principles of climate neutral economy and blue bioeconomy), digital (based on Industry 4.0 applications), and inclusive (ensuring that all citizens are actively engaged). Food production modernization (e.g., by implementing emerging technologies) and developing shorter and more domestic supply chains are also critical to achieving food resilience and security.

## 1. Introduction

We live in an era that future historians will refer to substantially due to the continuous dramatic changes in societal norms that occurred within 3-4 years. Initially, the alarming issue of climate change became a significant driver of political acts worldwide targeting a climate-neutral economy in the following decades. Successively, the global community was hit hard by the COVID-19 pandemic, which caused a humanitarian crisis with millions of deaths and simultaneously disrupted supply chains, food, and energy markets [1,2,3]. During the pandemic, the food sector faced numerous problems (e.g., an imbalance of supply and demand, disruption of the food distribution network, etc.) due to repeated lockdowns, revealing how fragile our food systems are [4,5]. The progress in population vaccination and advances in medicine and treatment development gave hope of recovering the socio-economic “normality” after a couple of years. However, the Russian–Ukrainian war shocked the world again. The war brought enormous casualties in both countries, triggering a “tsunami” of geopolitical and economic shifts globally [6]. It increased vulnerability and food insecurity worldwide, causing new challenges to the already volatile post-pandemic markets [7] and rendering even more complex supply management [8]. Only a few months after the beginning of the war, the living costs skyrocketed due to the ceasing of Ukrainian exports of essential agricultural commodities, strong global demand, new price spikes in foods, and questionable future harvests [9]. The post-pandemic fragile economies shattered further due to the vast economic sanctions imposed on Russia, which caused a domino effect on oil, energy, raw materials, and food prices, threatening to push millions of people into hunger and poverty [10].

The long-term effects of these crises on climate change mitigation and the transition to a carbon-neutral economy are constantly emerging and remain uncertain [11]. The nexus of climate change, pandemics, and wars (conventional, economic, trade, hybrid, etc.) are continuously pressing food, water, material, and energy resources [8,12,13]. The quantitative and qualitative depletion of the earth’s resources following climatic and geopolitical conflicts is accelerated by increasing contamination, environmental deterioration, and land use variations [6,14]. Likewise, this nexus puts at risk the agricultural sector [15,16], undermining the stability of global food systems and exposing vulnerable populations to undernourishment [17,18]. 

All these facts lead to the conclusion that systemic events are neither unlikely nor infrequent anymore, and disruptions are becoming the new norm in the world. It is now well established that extreme weather events will become more frequent, climate shifts will progress more, natural disasters will become more intense, and new pandemics will be unavoidable [19]. Moreover, the world population and urbanization are spreading rapidly, meaning more antagonism over soil and water resources and an ever-increasing food demand [1,15]. For instance, global food production must increase by at least 70% to feed the mammoth population of 10 billion people by 2050 [20,21]. This increase requires sustainable agriculture practices, concerted effort, and sustainability of the supply chain from farm to consumption [5,12,22]. The food-feed competition is an additional challenge, e.g., it is anticipated that over 1 billion tons of cereals will be used for animal feed, and the demand for animal products will increase by up to 70% by 2050 [23].

The current perspective article discusses how the food sector struggles to deal with multiple crises. Firstly, a literature review is conducted, investigating the consequences of the three consecutive crises and the proposed mitigation measures for each. The investigation focuses on articles published within the last 10–15 years regarding climate change effects, the previous three years regarding the pandemic, and the last year regarding the Russian–Ukrainian war. Then, the mitigation measures and recommendations found in different studies are grouped and classified on three levels (low, medium, and high) to describe their potential to increase the resilience and sustainability of food systems for the years to come.

## 2. The Impact of Climate Change on the Food Sector

The highly carbon-intensive (based on burning fossil fuel, coal, and gas) and unsustainable growth of humanity over the past two centuries has had dire repercussions for both the environment and the climate [24,25]. Climate change is defined as a long-term shift in weather patterns, as demonstrated by the unusual distribution of the mean temperature obtained over the last 30 years [26,27]. Indeed, the global atmospheric temperature is expected to rise by 4 °C by 2080. This temperature rise is mainly attributed to human activity doubling atmospheric carbon dioxide emissions [24]. Furthermore, these climatic shifts have resulted in an increased frequency of heat waves, droughts, winter storms, floods, and other extreme weather events [25].

The agricultural sector is an essential contributor to greenhouse gas (GHG) emissions, mainly caused by increased deforestation, intensive farming systems, livestock production, pastoral industry, and the excessive use of synthetic pesticides and fertilizers, aiming at higher production yields [15,16,28,29]. For example, the excessive use of synthetic N-fertilizers generates high nitrous oxide (N_2_O) emissions, while the production process of these materials is the cause of many GHG emissions [16,30,31]. Likewise, monocultural mass production with extensive fertilizer usage negatively impacts biodiversity and food quality [18,24]. Impacts on biodiversity raise concerns about the extinction of species that provide food for more than 60% of humans globally [32]. Besides, the rise of zoonotic diseases (including COVID-19) in recent decades has been partially attributed to biodiversity loss [25]. It is widely accepted that climate-induced shifts in rainfall patterns, temperature, sea water level, salinity, and nitrogen deposition devastate soil fertility, water availability, and crop productivity [15,33,34]. Indeed, climate change mainly impacts crop cycles and growth periods, and rain-depended cultivations, e.g., rice, maize, and wheat, are among the most negatively affected food crops over the last three decades [16,32]. More effects include food’s reduced nutrient content and availability of micro and macronutrients in the global food supply chain [18].

From another point of view, climate change affects the sources and modes of transmission, growth, and survival of food pathogens, as well as the origins and transmission routes. Subsequently, it affects the persistence and virulence of marine and freshwater algal blooms, parasites, fungi, bacteria, and vectors pathogenic to animals and plants. Extreme events substantially threaten food safety in the coming years by increasing foodborne infection rates and intoxication [34]. For instance, certain phenomena (e.g., flooding) can affect child growth long-term through the harshening of infectious diseases burden and modifications in food consumption [24]. Additional problems may arise from the growth of pests that increase food spoilage from farm to fork [18].

Besides, climate change substantially impacts fisheries [24] and current livestock systems throughout the supply chain, from production to processing, retailing, transport, storage, and consumption [35]. The aquatic food web is affected by ocean warming and alteration of the nutrient cycle and plankton production [36]. Raised temperatures and increased atmospheric carbon dioxide concentrations affect herbage growth for animals, while pest growth, disease outbreak, and water scarcity negatively affect feed, fodder, and livestock production [29]. The most significant impact can be felt in low-income countries and areas already vulnerable to food insecurity, generating food shortages, degrading the nutritional status of food, and causing adverse health outcomes on a long-term basis [24]. Besides, the increased intensity and frequency of extreme weather events pose a vital threat where access to mechanization and cooling systems is limited [35].

The ability of current livestock systems to support the increasing demand is also threatened because of the growing income of a large portion of the population in developing countries. In some countries, this demand has accelerated forestland burning in favor of crop production and pasture development to raise large ruminants [29,35]. In addition, many consequences of climate change threaten food quality, quantity, and equitable distribution, thus exposing vulnerable people in arid and semi-arid areas to malnutrition [16,18,33]. Furthermore, it can be anticipated that all food security components (availability, access, stability, and utilization) can be indirectly affected because of effects on incomes as well as health damages, especially in vulnerable populations [16]. For instance, climate change affects substantially small-scale farming in West Africa due to a lack of infrastructure, information gaps, environmental deterioration, and weak farmer organizations [37]. Finally, tangible and intangible traditional food systems are disrupted through frequent natural disasters due to decreased availability of local commodities, altered storage practices and food preparations, and reduced number of food festivals [24].

## 3. The Food Sector in the Post-Pandemic World

At the beginning of 2020, the COVID-19 pandemic outbreak and associated health and societal impacts caused a psychological shock and physical and economic disruption to markets and citizens [3,5]. The latter were unprepared for the sudden effects of this rare crisis on different sectors like raw material provision, tourism, and food value chains. In addition, social distancing limitations, curfews, and border and port restrictions reduced the competitiveness of vital productive sectors [38]. On a short-term basis, the pandemic accelerated domino effects on the food sector, including restaurant lockdowns, limited access to consumers, panic buying and scarcity of products on the shelves of groceries and supermarkets, food shortages and price spikes, reduced animal farming and health services in the livestock sector, immense labor loss, as well as increased food loss and waste from farm to fork [5,38,39]. Moreover, the pandemic caused population stress and mental health problems [40]. At the same time, it affected the eating habits and shopping behavior of consumers, who changed their purchasing methods and started to cook more than ever [5,41]. Other impacts of the pandemic on the food sector included reduced access to essential services, loss of income, lack of liquidity, and bankruptcy of many enterprises [3].

Long-term, four critical dimensions of food sectors were impacted: bioactive food compounds, safety, security, and sustainability [1]. Initially, the origin of coronavirus was linked to the wet market of Wuhan (China), while meat processing plants were referred to as suitable environments for outbreak onset. However, more recently, the possible virus transmission through the food chain has been questioned, leading to many food-safety lessons. For instance, food industries should apply more appropriate space management, food kitchens, and wet markets should be reorganized, and food workers and consumers should update their food hygiene practices [41]. But more importantly, the pandemic highlighted the importance of multilevel resilience in the food sector. Among the urgent challenges for the food industry after the pandemic outbreak was the development of affordable and sustainable products that enhance consumers’ health [5]. Indeed, the pandemic accelerated market mainstreaming of nutrient-rich foods (e.g., fruit, vegetables, cereals, spirulina, spices, etc.), nutraceuticals, supplements, immune-boosting diets, and products with a high concentration of bioactive compounds or products with an increased bioavailability of the nutrients they contain [42,43,44]. Besides, recent research focusing on the role of food in supporting human health and reducing dietary-related non-communicable diseases has also been accelerated [45]. For example, the development of nutritional products to help the health and immune system of consumers has become popular [3].

Nutrition and food security became the main drivers of the pandemic food systems and normative outcomes [46,47]. The main elements of food security (access, utilization, stability, and availability) were affected on a short-term basis and continue to be affected nowadays. Pandemic reverberations could exacerbate limited food access, food insecurity, global poverty, and hunger, affecting the poorest and most vulnerable populations [38] massively. For instance, food accessibility is threatened due to increased food costs, food distribution, infrastructure uncertainties, public transit access problems, interrupted global trade, and social inequities [48]. Subsequently, food sustainability and resilience are at stake due to several technological, economic, geopolitical, environmental, and social parameters that affect agriculture, food processing, and distribution. Food sustainability is tightly linked to food loss and food waste generated at any stage of the supply chain, e.g., during harvesting, processing, storage, retailing, transportation, and consumption. At the beginning of the pandemic outbreak, panic buying increased pressure on waste management systems and raised concerns about a rapid rise in food waste quantities. Likewise, after the repeated lockdowns, households are expected to waste more food, as consumers will have less time to cook at home [49]. On the other hand, the lockdown of billions of citizens during the pandemic outbreak and the repeated lockdown waves has accelerated the development of numerous innovations in the food sector, e.g., tools and apps to improve restaurant inventory management and reduce food waste [3]. The increasing pressure on regional food security has indicated the need for intensive sustainable food production systems (e.g., automation in smart agriculture) to mitigate the supply chain challenges from climate change and pandemics.

## 4. The Impact of the Russian-Ukrainian War on the Food Supply Chain

The ongoing Russian-Ukrainian war is the most prominent conflict in Europe since the Second World War, triggering numerous health, economic, and geopolitical implications [10]. The environment has been overlooked due to the extreme humanitarian crisis, but unquestionably, warfare activities of this scale cause substantial detrimental impacts on it [6]. Water quality and availability are affected by the destruction of industrial and public infrastructure, while continuous bombardment, troop movement, and potential radiation leakage increase greenhouse gas emissions and adversely affect air quality [6,10]. Moreover, explosions affect landscape morphology and cause soil degradation, altering its biological, chemical, and physical properties and thus destroying a carbon reservoir and vital resource for food production [50]. Ecosystem services (e.g., pollination, carbon sequestration, water purification, etc.) are also damaged by armed vehicle circulation and intense fights, causing deforestation and destroying urban green areas [51]. The violent destruction of habitats and deforestation also causes biodiversity loss and hinders ecosystems’ ability to contrast air pollution [6]. The damage will likely expand to regions beyond the battlefield and neighboring nations through shared rivers and ecosystems, while long-term impacts are expected to be irreparable [10].

Although there is never a good time for war, armed conflicts commenced during a disastrous period for global food markets since food prices were already high due to strong worldwide demand and the post-pandemic disruptions in the supply chain [9]. Moreover, conflicts occur in one of the world’s foremost “breadbaskets” [52]. For example, Ukraine and Russia play vital roles in the fertilizer markets [7]. At the same time, both countries supply 70% of globally traded sunflowers, 30% of wheat, and 20% of maize [53], with many countries from Africa, Asia, and the Middle East relying on Russia for affordable harvests [54,55].

More importantly, the current war does not represent only a regional conflict but a more resounding crack in Russia-West ties, with profound implications for geopolitics, the global economy, and the rest of the world [56]. The conflict has affected global energy markets and food security, resulting in fuel costs and food prices skyrocketing, threatening the global food markets, and having adverse implications for post-pandemic businesses [10,54]. An immediate consequence of the conflict has been the occurrence of food shortages in different countries due to the restrictions on international trade [6], as well as disruption to the feedstock supply chain and production of biofuels [7]. In addition, the food purchasing power of importing countries has been reduced, affecting the international food aid capacity to support countries (primarily low-income) highly dependent on purchases by bilateral and multilateral development agencies [57]. For example, the World Food Program buying around 50% of its grain from Ukraine, has been forced to restrict this ratio because of increasing costs. Finally, the ability of food systems to function correctly has been reduced by warfare activities, e.g., water infrastructure and agricultural fields are destroyed by military operations, supply chains are disrupted, harvest, process, and transport are impeded, production is reduced in the regions of the battlefield, and the ability of citizens and households to secure their food needs is weakened [58].

## 5. Targeting Food Sustainability and Resilience

**Figure 1:**
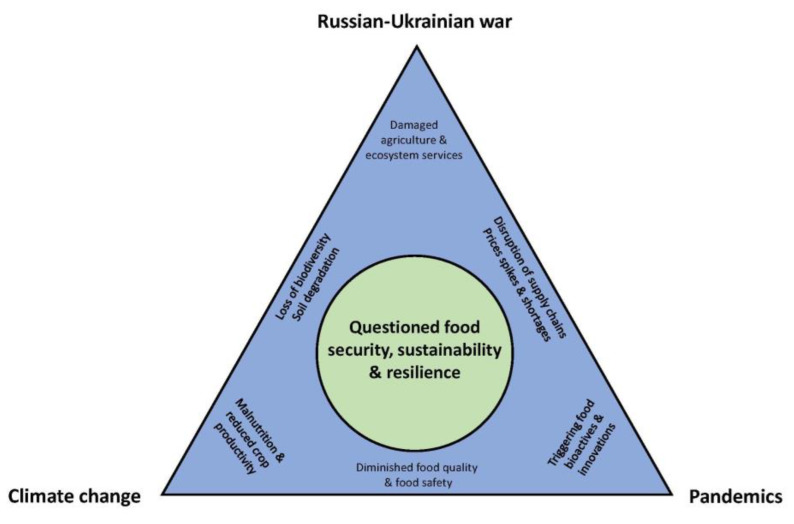
The impacts of climate change, pandemics, and Russian-Ukrainian war on the food sector.

Although different, crises like extreme weather events and natural disasters, pandemics, and war conflicts share common characteristics, e.g., they cause systemic shocks and disruption of markets and are non-stationary. Moreover, the mitigation measures taken for each one could address the impacts of the other one. The effects of multiple crises on food systems have attracted the attention of researchers, policymakers, and other actors, highlighting the need to revise the existing structures of the food sector [59]. Figure 1 illustrates the significant impacts of the three crises (climate change, pandemic, and Russian-Ukrainian war) on the food sector. Some effects concern only one type of crisis (placed in each corner of the triangle), and others are shared by two crisis types (placed on each side of the triangle). All these outcomes and implications lead to an important lesson (illustrated within the circle): crises generate food insecurity, and the food sector needs an urgent transformation towards food sustainability and resilience to adapt rapidly [3]. Both sustainable and resilient food systems could contribute to food security since they comprise complementary concepts [60,61]. Food resilience is the ability of food systems to maintain their goals by mitigating damages and absorbing disturbances.

In contrast, food sustainability is the ability of the supply chains to meet today’s demands without compromising future ones [62,63]. Table 1 presents several measures to tackle the impacts of the three different crises at three different mitigation levels (low, medium, and high). Generally, stabilizing food systems in a fast-changing world with inevitable problems is difficult. It can only be achieved by re-evaluating the system’s vulnerabilities, choke points, and weaknesses, and innovating critical services.

### 5.1. Mitigation Measures to Tackle the Impacts of Climate Change

There is a clear need to re-evaluate our policies, strategies, legal frameworks, and guidelines in the upcoming years to tackle the effects of climate change in the food sector, especially in the world’s regions with the most inadequate resources [14]. However, these efforts should consider human behavioral responses regarding food utilization, stability, access, and availability [16]. In addition, transparent and robust collaborations among all the relevant actors in the value chain are needed [64]. Mitigation measures should align with the United Nations Sustainable Development Goals (SDGs). They should be implemented in all food chain stages, e.g., in the supply end by following sustainable agricultural practices, in food distribution by improving food access, and in the consumption stage by reducing food demand and food waste [29]. The latter is critical, as food waste and loss generate 4.4 Gt CO_2_-eq annually, around 8% of anthropogenic GHG emissions [65].

The re-evaluation of food supply chains, minimizing external and internal disturbances, is also critical to ensure food security and sustainability and to reduce food waste [22]. A good strategy would be to focus on each country’s traditional and local staple foods [66,67]. Food supply chains of local foods are shorter and independent of third parties; thus, controlling the prices is more manageable [22]. Furthermore, food production should be intensified following community-based biodiversity conservation and sustainable forest management principles, using minimal land surface, restoring degraded land, and applying improved staking and integrated soil fertility solutions [32,68,69]. For instance, zero tillage is an agricultural practice that improves soil structure and biological diversity, enhances carbon sequestration and water efficiency, and reduces tillage machinery impacts, GHG emissions, and production costs [70]. Solutions are also needed for cultivating crops in regions with varying environmental conditions, such as variations in daylight duration. In some instances, air humidity variations and water stress could be mitigated by diminishing stomatal conductance without affecting the photosynthesis rate.

Furthermore, cultivation approaches that increase water productivity via irrigation are recommended. In contrast, cultivating biotic and abiotic stress-resistant crops and varieties across agroecologies is suggested to advance food security [32]. For example, higher temperatures and increased carbon dioxide concentrations are known to elevate the production yield of cassava [71].

The various crises indicate that we need a different systems thinking approach, e.g., increasing reliance on renewable resources and local energy systems to support the sustainable development of economies [11] and reducing the competition between biofuel and food markets in consuming the available agricultural resources [7]. This period comprises an essential opportunity to transform the economy into a sustainable bioeconomy and a more inclusive circular model that leaves no one out and promotes innovation. This approach requires the widespread and immediate adoption of policies targeting a climate-neutral economy [72]. Furthermore, valorizing high-diversity bioresources can achieve the noted goal, integrating biochemical and thermochemical processes and increasing the transition from first-generation biofuels to the production of higher-generation bio-based products [3,23,25,73]. Besides, it has been proved that the environmental benefit of utilizing bioenergy relies more on the soil’s carbon sequestration than the reduced GHG emissions of biodiesel or bioethanol [74].

The pasture-based food system and livestock production should also be optimized, e.g., by improving grazing management, selecting the correct breed, using grasslands efficiently, and improving reproductive efficiency [23,29]. The sustainability of livestock production systems requires consideration of the social dimensions and animal product consumption, adopting more sustainable diets based on alternative protein sources, and nutritious and healthy living [18,23]. These diets should be nutritious, adequately diverse, and better aligned with environmental conservation and contextual ecosystem functions [18]. Furthermore, we should increase genomic, genetic, and gene-editing resources for orphaned and current vegetables and fruits [75]. Plant-based foods instead of animal-based ones could feed hundreds of millions of people [57]. For example, 500 Gtn of seaweed could replace nearly 40% of the current soy protein production and, at the same time, absorb carbon. Indeed, they could sequester around 173 Mtn of carbon annually by exporting biomass to deep waters. Seaweeds also afford an excellent source of high added-value compounds with antioxidant, antiviral, and antimicrobial properties and other nutrients such as polyunsaturated fatty acids, fibers, and minerals [76].

On the other hand, governments should inform the general population using awareness and education campaigns on food safety risks linked to climatic factors [34]. Likewise, governments should invest in climate-resilient infrastructures (e.g., irrigation structures) and climate-friendly agriculture research [9,32]. At the same time, biorefineries and food systems should be decentralized to secure farmers, enterprises, and smallholders, e.g., by following the “biocities” model and applying smart specialization concepts. It is also vital to increase funding for innovations such as carbon farming, climate-smart forestry, food production automation with robotics, remote sensing, decision support systems, and big data analysis [3]. These innovations could enhance the ability of farmers to monitor food contamination, diseases, and pest spread while minimizing the usage of fertilizers; they could enable meteorologists to observe climate shift parameters and evaluate interactions among environmental factors and policymakers to develop comprehensive policies based on proper planning [9,77,78].

### 5.2. Mitigation Measures to Secure Food and Deal with Future Pandemics

One can argue that the pandemic has started a new era in how the food industry manages food safety, farm risks, working conditions, and system integrity. However, the global response was “reactionary” and not “preventative.” This approach is expected to be the same in future pandemics unless fundamental changes are made in the food chain and concerning how we consider and consume food. Firstly, adapting the “One Health” principles is necessary to control diseases that spread between animals and humans and minimize the risks of antibiotic resistance [3]. After that, the control of the pandemic spread and management of relevant outbreaks need synchronization, data sharing, and holistic risk assessment among several actors in the food supply chain, including epidemiologists, animal science researchers, the farming community, wet market traders, local businesses, exporters, and consumers [79].

Governments should develop preparedness plans (e.g., including critical data sharing among different ministries) for food safety incidents and natural disasters [34]. Mitigation measures include predictive models, intelligence gathering and foresight analysis, preparedness strategies, advanced monitoring mechanisms, and emergent removal of food contaminants [64]. They should also enhance communication strategies to secure consumers’ confidence in the safety of the agri-food system [5]. Besides, it is necessary to allow local communities to have a higher governance degree, e.g., to diversify distribution systems and logistic infrastructures support aiming at the partial re-territorialization of food systems [80]. Furthermore, governments and companies should focus on real-time food analysis and country-specific evaluations of threats and price shocks [9]. This goal can be achieved by developing an agri-food system that integrates logistics systems and information to trace back the delivery process and the entire production in case of food-safety incidents [81]. Likewise, supporting laboratories in developing early detection methods for pathogens is crucial. Tracing of foodborne illnesses and surveillance of pathogens can be implemented using automated and high-throughput genotype-based approaches (e.g., real-time PCR and next-generation sequencing) [34].

A critical reform of supply chains is also needed, e.g., matching consumers’ demands with shorter food supply chains to minimize uncertainties obtained by systemic risks [82], empowering all-food chain actors by implementing policies that emphasize their inclusiveness [34], futureproofing for the potential impacts of food security risks and intensifying food production systems through automation, smart agriculture, and “Industry 4.0” applications. Relevant innovations (e.g., blockchain technology, Artificial Intelligence, and the Internet of Things) may be boosted by services dealing with digitization and Internet and Communication Technologies using meteorological data linked with climate modeling [83]. Well-developed irrigational systems, new crop cultivars, optimized input usage, and other green revolution measures should become a priority [16,84,85,86]. All these actions need training of the workforce in emerging technologies, robotics, and disruptive technologies, using modern education techniques. For example, virtual accelerator hubs for in situ and remote end-user innovations could assist micro, small, and medium enterprises overcome hurdles that may come with training and networking in meeting rooms. Vertical farming, rooftop gardening, and, more generally, urban agriculture could contribute to green recovery by reducing dependency on longer supply chains and boosting consumers’ education in agroecological practices [3].

Finally, innovative products such as lab-grown meat, plant-based meat alternatives, foods developed by synthetic biology and precision fermentation, development of immune- and health-boosting products based on target food bioactive ingredients, are also becoming popular with Millennials and Generation X, along with changes in eating habits and personalized nutrition [3,5,13]. In addition, valorizing sources could drive the recovery of bioactive food like food processing by-products, mushrooms, yeasts, fungi, seaweeds, and algae. These solutions, which cope with bioeconomy and climate-neutral economy policies, can increase food security in the future and, at the same time, mitigate the impacts of climate change [1,3,87,88,89,90,91].

### 5.3. Mitigation Measures to Deal with the Impacts of Geopolitical Conflicts

The challenges facing the food sector as a result of geopolitical and armed conflicts are typically addressed by the international community with fast temporary measures such as enhancing food transfers, ensuring food assistance, establishing a strategic food reserve mechanism, and, most importantly, encouraging the respect of water- and food-related activities and infrastructure, with appropriate sanctions in case of violations [92]. Although these measures are always necessary, they only provide short-term solutions. In addition, sanctions against other countries and relevant tools (e.g., the carbon border adjustment mechanisms) cannot ensure energy and food resilience [93]. For example, the European Union (EU) is trying to reduce gas imports from Russia to increase energy independence, while policymaking worldwide is looking for new fossil fuel supply routes [94]. As a result, the gas and oil industries will become even more robust, generating new lock-ins, and the economies will lose an opportunity for climate-friendly energy transitions [11]. Therefore, if future economies are based on using non-renewable energy, governments must continue to prefer natural gas to oil. However, the current energy crisis has made us forget that natural gas is a fossil fuel with all its disadvantages regarding biodiversity loss and environmental and health implications. Contrarily to natural gas, hydroelectric and renewable energy consumption is known to decrease GHG emissions in the short and long run [95].

Besides, the current production trends promoting energy-dense ultra-processed foods are under scrutiny [96]. Subsequently, the food sector requires a transformation with target policies that protect agricultural areas, reduce pesticide and fertilizer use, and increase organic farming [5]. The food price crisis of the previous decade (2007–2008) shows that countries should avoid implementing sanctions restricting food, fertilizer, or commerce stockpiling. Further mitigation measures in this direction include providing subsidies or implementing a lower tax policy for fertilizers and energy targeting SMEs and farmers. It is also critical to enforce policies that lessen dependency on a few exporting countries and substitute maize and wheat with local and traditional crops [9]. Furthermore, the current global trade model should be renewed, e.g., just-in-time distribution chains and cheaper imports should be replaced with domestic production and storage [57]. Indeed, local and traditional foods should become a priority to close the urban-rural gap regarding energy savings from transportation expenditure [5].

## 6. Conclusions

The pressing challenges induced by climate change, global warming, the COVID-19 pandemic, and the Russian-Ukrainian war merge to conclude that the food sector needs an urgent transformation toward sustainability and resilience. To achieve this goal, all relevant actors (e.g., governments, companies, multilateral organizations, donors, farmers, etc.) within the supply chain should play their role by designing and implementing target interventions and policies. The transformation of the food sector should be inclusive, ensuring that all citizens are actively engaged, and no one is left out. In addition, this transformation is linked to the transition from fossil-based fuels and linear economy towards biobased products and a climate-neutral economy, respectively. Digital transformation and food production modernization are also needed, e.g., by implementing emerging technologies and Industry 4.0 applications at all stages of the food chain. The reduction of food loss and food waste, as well as the valorization of a vast range of bioresources, utilizing food processing by-products and highlighting “blue bioeconomy” (e.g., the development of multitrophic systems, seaweeds, and microalgae cultivation, etc.) can support food security. Governments should allocate resources for agroecological research that minimizes external inputs (e.g., pesticides), while actors in the food chain should be proactive regarding fresh food safety. It is also vital to promote domestic food systems, targeting the development of multiple shorter supply chains based on seasonal and traditional products. Consumers should redefine how they consume energy, goods, and food and become the driving force of the noted transformations. Finally, more studies and investigations are needed to expand and validate the stated mitigation measures.

## Figures and Tables

**Table 1:** Three different levels (“+” for low, “++” for medium, and “+++” for high) of mitigation measures to tackle the food sector challenges induced by various crises.

Mitigation Measure	Climate Change	Pandemics	Geopolitical Conflicts
Transparent and robust collaborations among all the relevant actors	++	+	+++
Synchronization, data sharing, and holistic risk assessment	++	+++	+++
Preparedness strategies and advanced monitoring mechanisms	++	++	+++
Sustainable agricultural practices	++	+	+
Improvement of food access	+		+++
Reduction of food demand and food waste	++	+	+
Decentralization of agricultural systems and shortening of supply chains	++	+++	+++
Focus on local and traditional foods	++		++
Enhancement of food transfers and food assistance		+	++
Diversification of distribution systems and logistic infrastructures	+	++	++
Supporting of laboratories to develop early detection methods for pathogens		+++	
Intensification of food production systems through automation, smart agriculture, and Industry 4.0 applications	+	++	+
Training of the workforce in emerging technologies, robotics, and disruptive technologies	+	++	+
Climate-resilient infrastructures	+++	++	+
Optimization of livestock production	++		+
Increasing reliance on renewable resources and local energy systems	+++		++
Ιncreasing genomic, genetic, and gene-editing resources	+	+	
Adaptation of “One Health” principles	++	+++	
Transition to circular economy	+++		
Valorization of food waste and diverse bioresources	+++	+	++
Sustainable diets based on alternative protein sources	+++	+	+
Awareness and education campaigns on food safety risks	+	++	+
Development of immune- and health-boosting products based on target food bioactive ingredients		++	

## Data Availability

All data used in this study is publicly available at databases (Scopus, Web of Science, ScienceDirect etc.).
